# Language impairment in a case of a complex chromosomal rearrangement with a breakpoint downstream of *FOXP2*

**DOI:** 10.1186/s13039-015-0148-1

**Published:** 2015-06-10

**Authors:** Daniela Moralli, Ron Nudel, May T. M. Chan, Catherine M. Green, Emanuela V. Volpi, Antonio Benítez-Burraco, Dianne F. Newbury, Paloma García-Bellido

**Affiliations:** Wellcome Trust Centre for Human Genetics, Roosevelt Drive, Headington, Oxford, OX3 7BN UK; Faculty of Linguistics, Philology and Phonetics, University of Oxford, Walton Street, Oxford, OX1 2HG UK; Worcester College, University of Oxford, Oxford, OX1 2HB, UK; Department of Biomedical Sciences, University of Westminster, 115 New Cavendish Street, London, W1W 6UW UK; Faculty of Modern languages, University of Oxford, 47 Wellington Square, Oxford, OX1 2JF UK; Department of Spanish Philology and its Didactics, University of Huelva, Huelva, Spain

**Keywords:** Language impairment, Chromosomal rearrangement, *FOXP2* regulation, Non-coding elements, Spanish

## Abstract

**Background:**

We report on a young female, who presents with a severe speech and language disorder and a balanced *de novo* complex chromosomal rearrangement, likely to have resulted from a chromosome 7 pericentromeric inversion, followed by a chromosome 7 and 11 translocation.

**Results:**

Using molecular cytogenetics, we mapped the four breakpoints to 7p21.1-15.3 (chromosome position: 20,954,043-21,001,537, hg19), 7q31 (chromosome position: 114,528,369-114,556,605, hg19), 7q21.3 (chromosome position: 93,884,065-93,933,453, hg19) and 11p12 (chromosome position: 38,601,145-38,621,572, hg19). These regions contain only non-coding transcripts (ENSG00000232790 on 7p21.1 and TCONS_00013886, TCONS_00013887, TCONS_00014353, TCONS_00013888 on 7q21) indicating that no coding sequences are directly disrupted. The breakpoint on 7q31 mapped 200 kb downstream of *FOXP2*, a well-known language gene. No splice site or non-synonymous coding variants were found in the *FOXP2* coding sequence. We were unable to detect any changes in the expression level of *FOXP2* in fibroblast cells derived from the proband, although this may be the result of the low expression level of *FOXP2* in these cells.

**Conclusions:**

We conclude that the phenotype observed in this patient either arises from a subtle change in *FOXP2* regulation due to the disruption of a downstream element controlling its expression, or from the direct disruption of non-coding RNAs.

**Electronic supplementary material:**

The online version of this article (doi:10.1186/s13039-015-0148-1) contains supplementary material, which is available to authorized users.

## Background

Developmental language disorders provide a window into the biological underpinnings of language [1, 2]. The characterization of clinical cases with genetic anomalies that can be associated with (endo)phenotypes of language is helping to unravel the genetic pathways underlying this human ability. One of these genes is *FOXP2*, a transcription factor located in 7q31 [3]. A missense mutation in *FOXP2* was first identified in a family (KE) with orofacial dyspraxia and language deficits affecting lexical semantics, morphology, syntax, and phonology [4–7]. Broad cognitive deficits were also observed in affected members [5, 6].

Subsequent studies have described different disruptions of *FOXP2*. Balanced translocations involving 7q31 have been described, both directly affecting the coding region of *FOXP2* [8] or with breakpoints near the gene [9]. They normally give rise to speech and language impairments, possibly in the form of spastic dysarthria [8], language deficits [10] and severe speech impairment [9]. More complex cases carrying mutations and microdeletions of *FOXP2* have also been described [11–13].

In this paper, we report on a young female who presents with a severe speech and language disorder and a *de novo* chromosomal rearrangement involving chromosomes 7 and 11. Given the phenotype of this patient and the karyotypic profile [14], we hypothesized that she may represent an additional *FOXP2* case. We used molecular cytogenetics to map the chromosome breakpoints and discovered a complex rearrangement involving an inversion of chromosome 7, followed by a translocation between the inverted chromosome 7 and chromosome 11 {(46, XX, der(7)inv(7)(p15;q31)t(7;11)(q21;p12), der(11)t(7;11)(q21;p12)}. The precise localization of the 7q31 breakpoint was further refined by PCR analysis, and SNP- and additional sequence-based analyses were performed.

## Results

### Clinical history

The patient was born after 42 weeks of gestation to a 27 years and 10 month old female. No complications were observed during the pregnancy and the delivery was normal. At birth, the weight was 2.550 kg, the height 50.5 cm and cephalic perimeter 35 cm. APGAR evaluation scores were normal. A moderate neonatal depression was observed in the newborn prompting the administration of intravenous fluids. Further exploration suggested intrauterine malnutrition, moderate jaundice without hepato-splenomegaly and vaginal bleeding. The child had feeding difficulties with frequent vomiting episodes but motor milestones were normal. Binocular astigmatism was present, requiring the use of glasses. An audiometry performed at 11 years and 2 months, revealed 10 % hearing loss in the right ear and a 5.4 % hearing loss on the left ear.

### Language and neurodevelopment

The proband languages are Castilian-Spanish and Valencian. She first spoke at 12 months of age but her expressive language was severely delayed and articulation imprecise. At 5 years of age, she used only sequences of two words and her speech production was unintelligible. Velar stops, alveolar rhotics and laterals were not produced. Nasal and fricative alveolar articulations were not present after vowels in consonant clusters. There were frequent substitutions, omissions and miss-timings of single articulations in a sequence. EEG analysis showed normal activity (63/04) at this age. She has attended speech therapy sessions since the age of 5. The Illinois Test of Psycholinguistic Aptitude [15] was administered at 9 years and 6 months of age and the proband was found to score below typically developed children (Additional file 1: Table S1). In motor expression the proband scored three years below expected.

At age 9 years and 11 months, she was diagnosed with Specific Language Impairment. At age 10 years and 8 months, her total IQ score [16] was in the normal-low range (88), her verbal IQ was low (74) while her non-verbal IQ was above the mean for her age (106). Verbal deficits were particularly prominent in the information and vocabulary subtests in both languages (Additional file 1: Figure S1 A, B). A laterality test pointed to left handedness but right visual preference. The proband shows empathy to others and socializes with friends, usually of a younger age. She displays impulsive behavior but shows explicit attempts to resolve conflict with others. At the onset of puberty (age 12 years, 11 months), the proband showed sleep disturbance and symptoms of Obsessive Compulsive Behavior. Medication was initiated at 13 years and 3 months (a serotonin reuptake inhibitor), improving sleep patterns and obsessive-behavior. However, at age 15 years and 1 month, she reported auditory hallucinations. Additional medication (quetiapine hemifumarate) was started at age 15 years and 2 months, and the auditory hallucinations seem to have stopped. The difficulties experienced by the proband led to problems following a normal pace of learning at school and she has attended a special education unit since the age of 13 years and 9 months.

None of the patient’s family members have suffered developmental language disorders from the maternal or paternal side.

### Sequencing of the coding regions of *FOXP2*

In order to rule out the involvement of *FOXP2* by incidental mutation, we sequenced the coding regions of all *FOXP2* transcripts. No splice site or coding variants were found (data not shown).

### Molecular cytogenetic analysis

**Fig. 1:**
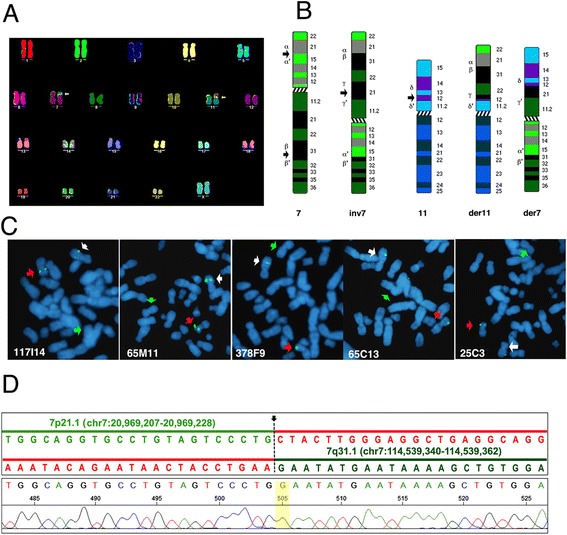
**a** MFISH karyotype from the proband. The arrows point to the chromosome 7–11 translocation. **b** hypothetical steps involved in the proband’s rearrangement. Each of the putative breakpoints is identified by Greek letters. Breakpoints in 7p (α/α’) and 7q (β/β’) lead to a pericentric inversion of chromosome 7 to generate an inv7 intermediate. This was followed by a translocation between the inv7 p (γ/γ’) and 11 p (δ/δ’). **c** FISH with BACs (green signals) spanning the *FOXP2* locus, on metaphase spreads from the proband. Each panel shows the hybridization signal from a single BAC probe, as indicated. The chromosomes are counterstained in DAPI, blue. The five BACs map to the normal chromosome 7 (white arrow), and to the derivative chromosome 11 (red arrow). No signal is found on the derivative chromosome 7 (green arrow). **d** Chromatogram of PCR mapping of the inversion breakpoint, with reference genome (GRCh37/hg19) sequences shown atop and breakpoint marked with a black arrow and dotted line. The sequence preceding the highlighted nucleotide “G” is on 7p21.1 (light green); the sequence following is on 7q31.1 (dark green). Segments in red show the respective continuing and preceding sequences on 7p21.1 and 7q31.1 in the reference genome

A complex chromosomal rearrangement in this proband had been previously suggested by classical cytogenetic analysis [14]. M-FISH (Fig. 1a) and DAPI banding on metaphase spreads from peripheral blood lymphocytes confirmed the presence of a 7 pericentric inversion, and a 7–11 reciprocal translocation. We therefore hypothesized a rearrangement involving two steps: firstly a pericentric inversion of chromosome 7 with one breakpoint around 7p15 and one around 7q31 (Fig. 1b, α/α’ and β/β’ respectively), which generated a lost intermediate inverted chromosome 7 (Fig. 1b, inv7). This event was followed by a reciprocal translocation between the inv7 and the short arm of chromosome 11, with breakpoints around 7q21 and 11p12 (Fig. 1B, γ/γ’ and δ/δ’ respectively).

Considering that *FOXP2* is localized in band 7q31 (114,055,052-114,333,827, hg19) possible disruption of its locus was probed using five BACs (Table 1) spanning region 113,695,289-114,374,043 (hg19), on chromosome 7. The BACs mapped correctly to chr7q31 in the parents. In the proband, the five BACs were found to hybridize to the derivative chromosome 11 short arm or der(11)t[7q31;11p12], in addition to the normal chromosome 7 as expected (Fig. 1c). These results indicate that although the *FOXP2* locus has been translocated to derivative chromosome 11, the locus itself was not directly disrupted by the breakpoint, which is located distally of position 114,374,043 (hg19).

**Table 1:** Results of FISH on metaphase spreads from the proband

7p breakpoint (α/α’)				
BAC	Start (hg19)	End (hg19)	Cytogenetic band	Localization in Proband
RP11-61 N24	20,748,162	20,919,479	7p21.1-p15.3	Derivative 11p
RP11-799 L23	20,810,041	21,001,537	7p21.1-p15.3	Split: derivative 7q, derivative 11p
RP11-97 L4	20,954,043	21,104,475	7p15.3	Split: derivative 7q, derivative 11p
7q breakpoint (β/β’)				
BAC	Start (hg19)	End (hg19)	Cytogenetic band	Localization in Proband
RP11-259A16	114,374,055	114,528,369	7q31.1-31.2	Derivative 11p
RP11-1 N24	114,451,504	114,603,837	7q31.1-31.2	Split: derivative 7q, derivative 11p
RP11-243D16	114,556,605	114,733,501	7q31.1-31.2	Derivative 7q
RP11-259A16	114,374,055	114,528,369	7q31.1-31.2	Derivative 11p
7q Breakpoint (γ/γ’)				
BAC	Start (hg19)	End (hg19)	Cytogenetic band	Localization in Proband
RP11-7B9	93,714,463	93,884,065	7q21.3	Derivative 7p
RP11-248I13	93,933,453	94,094,489	7q21.3	Derivative 11p
11p breakpoint (δ/δ’)				
BAC	Start (hg19)	End (hg19)	Cytogenetic band	Localization in Proband
RP11-63D14	38,455,222	38,604,308	11p12	Derivative 7p
RP11-81O6	38,601,145	38,749,858	11p12	Split: derivative 7q, derivative 11p
RP11-99H2	38,621,572	38,794,051	11p12	Derivative 11p

To localize the putative breakpoints within each chromosome region, we hybridized a series of genomic BAC clones (Table 1 and Additional file 1: Table S2) on metaphase spreads from peripheral blood and skin fibroblasts from the proband, and parents. The hybridization pattern of all BACs was normal in one copy of the proband’s chromosomes 7 and 11, and in both chromosomes in the parents (data not shown), confirming that the rearrangement was *de novo*.

#### 7p21-15 (α/α’ inversion breakpoint)

The location of the 7p inversion breakpoint was defined by four overlapping BACs (RP11-61 N24, RP11-799 L23, RP11-97 L4, and RP11-1129E15 - Table 1). In the proband, RP11-61 N24 localized in the derivative 11p, RP11-799 L23 localized partially on the derivative 11p and partially on the derivative 7q, RP11-97 L4 localized partially on the derivative 11p and partially on the derivative 7q and RP11-1129E15 localized in the derivative 7q (Additional file 1: Figure S2). Based on these data, the breakpoint maps to the region shared by BACs RP11-799 L23 and 97 L4, chr7:20,954,043-21,001,537 (hg19, 47.5Kb) on 7p21.1-15.3. This region contains a long non-coding transcript (AC006481.1 or ENSG00000232790).

#### 7q31 (β/β’ inversion breakpoint)

Three overlapping BACs (RP11-259A16; RP11-1 N24; RP11-243D16) located the inversion breakpoint on chromosome 7q31 (Table 1, Additional file 1: Figure S2). In the proband, the signal from RP11-259A16 was localized on the derivative 11p only, while that from RP11-1 N24 was split between the derivative 11p and the long arm of t(7,11) (henceforth called derivative 7), and the signal from RP11-243D16 was found on the derivative 7q only. Thus, the 7q breakpoint is likely to be in the region that is unique to RP11-1 N24, not shared by either RP11-259A16 or RP11-243D16. This corresponds to chr7:114,528,369-114,556,605 (hg19, 28Kb). This region contains no known genes.

#### 7q21.3 (γ/γ’ translocation breakpoint)

The second 7q translocation breakpoint was mapped to band 7q21.3, using BACs RP11-7B9 and RP11-248I13 (Table 1). RP11-7B9 localized to the derivative 7 short arm, and RP11-248I13 to the short arm of the derivative 11 (Additional file 1: Figure S2). Thus, the breakpoint maps in the region between these two BAC probes, chr7:93,884,065-93,933,453 (hg19, 49Kb) on 7q21.3. This region contains lincRNAs (large intergenic non coding RNAs) or TUCPs (transcripts of uncertain coding potential), TCONS_00013886, TCONS_00014353, TCONS_00013888.

#### 11p12 (δ/δ’ translocation breakpoint)

Finally, we located the breakpoint on chromosome 11 using three overlapping BACs: RP11-63D14, RP11-81O6 and RP11-99H2 (Table 1). RP11-63D14 was localized on the derivative 7 short arm, the RP11-81O6 signal was split between the derivative 7 short arm, and the derivative 11 short arm, and RP11-99H2 mapped to the derivative 11 short arm (Additional file 1: Figure S2). Thus, the breakpoint is localized within the region unique to BAC RP11-81O6, chr11:38,601,145-38,621,572 (hg19, 20.4 Kb). This region contains no known genes, or non coding elements.

In summary, the FISH data support the hypothetical two-step model. The hybridization pattern of all BAC probes was identical in peripheral blood cells and fibroblasts, implying that the rearrangement is not present as mosaic in the patient.

*FOXP2* is not directly affected: the FISH of five BACs spanning its locus showed that it moved to the derivative 11p, while the region distal to the gene remained in 7q (Table 1, Fig. 1c). In conclusion, the only transcripts potentially split by the translocation or inversion events were ENSG00000232790 (7p21.1-15.3) and TCONS_00013886, TCONS_00013887, TCONS_00014353, TCONS_00013888 (7q21.3).

Affymetrix Cytoscan HD arrays analysis in the parents and proband identified no obvious loss of material in the regions around the predicted breakpoints. CNV analyses identified only three inherited copy number variants in the proband, none of which coincided with the *FOXP2* locus (Additional file 1: Figure S3).

### PCR breakpoint locus analysis

The breakpoints of the inversion event were precisely mapped by PCR analysis using primers localized in the 7q31 and 7p21.1-15 regions. A nested Sanger sequencing reaction produced a fragment that contained sequences mapping to chr7p21.1(−) and chr7q31.1(+), allowing us to determine the inversion breakpoints to be chr7: 20,969,207 and chr7:114,539,340, respectively (hg19, Fig. 1d). The chromosome 7q breakpoint position is 205,513 bp from the 3′ end of *FOXP2* and 22,868 bp from the 5′ of *MDFIC*. The chromosome 7p breakpoint directly disrupts the lncRNA ENSG00000232790.

### Expression analysis

We used Affymetrix gene expression arrays to compare the expression levels of genes in and around the mapped breakpoints in the parents, a sibling and the proband, on RNA extracted from primary skin fibroblasts (LIMMA PLIER analysis, Additional file 2: Table S5). Two of the non-coding transcripts that fell within the predicted breakpoints had probes included on these arrays (ENSG00000232790 on chromosome 7p21, and TCONS_00013886 on chromosome 7q21). Neither showed significant expression differences between the sister and the proband (*P* = 0.91 and *P* = 0.99 respectively). Notably, no significant differences were seen for the expression level of *FOXP2*, although the expression levels of *FOXP*2 and the non-coding transcripts were all too low to accurately assess differences between samples. However, the expression of some of the genes controlled by *FOXP2* was altered in the proband compared to the sibling. For example, *EFNB2*, *INHBB*, *NTN4*, *ROBO2*, and *SLC14A1* were upregulated > 3 fold in the proband (LIMMA PLIER analysis, Additional file 2: Table S5). *FOXP2* expression was therefore further analyzed by Taqman assay, using two probes. Again, expression levels were low and inconsistent across RNA batches, precluding a meaningful comparison between the proband, sibling and parents (Additional file 1: Table S3 and Additional file 1: Table S4).

## Discussion

In this paper, we use molecular cytogenetics to localize the breakpoints in a young female who presents with a severe speech and language disorder and a *de novo* chromosomal rearrangement involving chromosomes 7 and 11. We report a complex rearrangement involving an inversion of chromosome 7, followed by a translocation between the inverted chromosome 7 and chromosome 11 {(46, XX, der(7)inv(7)(p15;q31)t(7;11)(q21;p12), der(11)t(7;11)(q21;p12)}. The breakpoint on 7q31 was found to map 200Kb from the 3′ end of *FOXP2* and 22Kb from the 5′ of *MDFIC* and directly disrupts the lncRNA ENSG00000232790. The precise location of the addition breakpoints have yet to be determined but the critical regions contain no coding transcripts. We examined the expression levels of genes around each of the putative breakpoint sites but did not find any evidence for altered gene expression in these regions.

The breakpoint on 7q31 maps 200Kb to the 3′ UTR of *FOXP2*, in which several microRNA target sites have been described. Although the breakpoint is a distance from this region, it may have disrupted other yet unknown expression-controlling elements. Interestingly, we did observe that the expression of some *FOXP2* targets was altered in the proband compared to her sister. Among them, *ROBO2* is involved in thalamocortical axons development [17] and has been related to autism and asocial behavior [18], but also to dyslexia and expressive vocabulary growth in the normal population [19]. Although this may represent an artefact of the analyses (e.g., differences due to age or gender effects), we cannot rule out the possibility that the expression pattern of *FOXP2* has been affected. Further experiments will be required to confirm this hypothesis, but at least one other similar patient has been previously described [9] with breakpoints localized 500 kb downstream of *FOXP2* UTR region.

We did observe direct disruption of a non coding element at the 7p21.1-15.3 breakpoint, which might account for the linguistic deficits observed in the proband. As it is impossible to assess gene expression directly in the brain, we chose to examine gene expression levels in primary skin fibroblasts which express several neuronal specific receptors and enzymes [20]. However, this approach does assume that gene expression levels can be generalized across tissues. Although no expression differences were found in ENSG00000232790 and TCONS_00013886 between samples, further validation would be needed to assess the functionality of these transcripts. Long non-coding RNAs are important for fine-tuning of gene regulation and brain development [21]. They have been implicated in neurodevelopmental disorders [22]. Developmental delay caused by a disruption of a lincRNA as a result of a translocation has also been observed [23]. These studies, as well as our own open up new avenues of research into the potential involvement of non-coding RNAs in language disorders. Further additional analyses involving alternative routes will be required to confirm whether the observed genetic changes or other factors contribute to the language impairment in this individual.

## Conclusions

In conclusion, the exact genetic cause of the language impairment exhibited by this clinical case remains to be fully elucidated. Crucially, a better understanding of the role played by non-coding sequences in regulating brain development may help to understand this complex dysfunction.

## Methods

Ethics approval for this research was granted by the University of Oxford [MSD-IDREC-C1-2012-95, SSD/CUREC2/09-23].

### Fluorescence in-situ hybridization

Metaphase spreads were harvested from peripheral blood or cultured skin fibroblasts using standard techniques [24]. The MFISH was carried out with the 24XCyte kit (Metasystem, Zeiss LTD) according to manufacturer instructions. Genomic BACs were a kind gift from Dr Sam Knight, the Sanger Institute, or obtained from Source Bioscience and Invitrogen. Probes were labelled by incorporating biotin-16 dUTP, digoxin-11 dUTP, or FITC-16 dUTP (Roche) with the Nick Translation Kit (Abbot Molecular) following the manufacturer instructions. FISH was performed using standard techniques [25]. Slides were counterstained and mounted in DAPI/Vectashield (Oncor) and analysed with an Olympus BX60 microscope for epifluorescence, equipped with a Sensys CCD camera (Photometrics, USA), using Genus Cytovision software (Leica).

### PCR breakpoint locus analysis

To refine the inversion breakpoint on chromosome 7q31, long-range PCRs were performed using Phire Hot Start II DNA polymerase (Thermo Scientific) to amplify fragments covering the breakpoint locations, as determined by the cytogenetic analysis. Primers were designed within Primer 3 [26] using a reference genome (hg19). Both forward and reverse primers were designed so that they would bind to the same strand on the reference sequence, as following the inversion the plus and minus strands from both sides of the breakpoint are connected (TCATGCAATGTGTCCCCAAA, GATTTGCTTAACTGCCCTGC). Thus PCR products would only be generated if the fragment contained the breakpoint. The exact breakpoint was then mapped through a nested Sanger sequence reaction, with internal primers covering the length of the sequence predicted to be contained in the PCR product (GACTATTTCCAGCCTCTTTATCCT). These were the primers for the product that contained the breakpoint, and that other primer sequences are available upon request.

### Mutation screen

The coding regions of *FOXP2* were amplified with the primers used by McDermot *et al.* [11] via PCR with a 1:9 ration of PFU (Thermo Scientific) and BIOTAQ (Bioline) and 40 ng of DNA from the proband, extracted from peripheral blood. The fragments containing the coding regions were sequenced using Sanger sequencing.

### Affymetrix SNP arrays for Copy Number Variation (CNV) analysis

DNA samples (250 ng extracted from lymphocytes) from the parents and proband were genotyped on Affymetrix Cytoscan arrays by AROS Applied Biotechnology (www.arosab.com). These arrays include 750,000 SNPs and allow the detection of copy number changes and chromosome aberrations in addition to genotypic information. SNP data were analysed by Affymetrix Chromosome Analysis Suite (ChAS) and each of the regions surrounding the identified breakpoints examined for loss or duplication of material, which would be represented by changes in Log2Ratio and allele peaks.

### Gene expression analyses

Total RNA was extracted from fibroblast cell lines generated from skin biopsies from the proband, her sister and her parents, and was isolated using the Qiagen Mini RNeasy kit following the manufacturer’s instructions. RNA integrity was assessed on a BioAnalyzer (Agilent Technologies). 200 ng RNA from each sample in three replicates was used to generate labelled sense single stranded DNA (ssDNA) for hybridization, with the Ambion WT Expression Kit, the Affymetrix WT Terminal Labelling, and Controls Kit and the Affymetrix Hybridization, Wash, and Stain Kit as described by the manufacturer. Sense ssDNA was fragmented and the distribution of fragment lengths was measured on a BioAnalyzer. Fragmented ssDNA was then labelled and hybridized to the Affymetrix GeneChip Human Gene 2.0 ST Array (Affymetrix). Chips were processed on an Affymetrix GeneChip Fluidics Station 450 and Scanner 3000.

Affymetrix CEL files were RMA normalized in GeneSpring GX 12 and differentially expressed genes identified using Limma, with a Benjamini and Hochberg multiple testing correction and a p-value cut off of ≤0.05. A fold change difference of ≥1.5 was used. In order to investigate if the expression of genes around the putative breakpoint sites or genes which are known to be targets of *FOXP2* would show any significant deviation in the proband from the family, the expression of these genes was assessed using LIMMA PLIER. Average expression levels were first compared across replicates between the proband and her sister. Genes which had significantly different expression levels in the proband and her sister were then manually compared between the parents and the proband.

In addition, Taqman probes were used to specifically measure the expression levels of *FOXP2* transcripts in fibroblast cells from the proband and her family. 0.5 μg of RNA was reverse transcribed using the QuantiTect Reverse Transcription kit (Qiagen) following the manufacturers protocol. Three RNA batches were used. TaqMan assays from Applied Biosystems (Life Technologies) were used to perform duplex qPCRs using the standard TaqMan protocol [27]. Two *FOXP2* probes were used: Hs01074134_m1 for the detection of transcripts NM_148899.3, NM_148900.3, NM_014491.3, and NM_001172766.2, and Hs01081804_m1 for the detection of transcripts NM_148898.3 and NM_001172767.2, both of which were FAM-labeled. Mean expression levels (across triplicate samples, and RNA batches) were determined using the ∆∆CT method [28] to compare *FOXP2* expression levels between the proband and her family members. Outliers in the CT readings were removed from the gene expression analyses. Gene expression levels were normalized against the endogenous control gene *IPO8* (TaqMan probe Hs00183533_m1, primer-limited, VIC-labeled).

## Additional files

Additional file 1:
**Table S1.** Proband scores at 9;6 (9 years and 6 months of biological age) for The Illinois Test of Psycholinguistic Aptitudes. **Table S2.** All BACs hybridized to patient sample to map breakpoint location. The BACs localization in the patient is highlighted in color (e.g., the derivative 11p in blue, and the derivative 7p in yellow). **Table S3.** Average C_T_ values across three RNA batches. In parentheses, SE, standard error. **Table S4.** Relative expression levels of *FOXP2* in all family members compared to the proband, measured with Taqman probes. **Figure S1.** A Typical Scores of the proband aged 10;8. Subtests where answers were required using language are classified as Verbal. Subtests where language was not required to provide answers are marked Manipulative. Typical Scores within 8-12 are normal. B. Typical Verbal Scores of the proband. Typical Verbal Scores refer to subtests where answers are required in Castilian-Spanish or Valencian. Typical Scores within 8-12 values are normal. **Figure S2.** FISH on metaphase spreads from the proband. Each panel shows the hybridization signal (green) from a single BAC probe, as indicated (the library name has been omitted). The chromosomes are counterstained in DAPI, blue. In each panel a white arrow identifies the normal chromosome 7 (where present), a green arrow the derivative 7, a yellow arrow the normal chromosome 11 (where present), and a red arrow the derivative 11. **Figure S3.** CNVs were mapped in the probands and parents using Affymetrix Cytoscan and copy number changes were called within Affymetrix Chromosome Analysis Suite (ChAS). Stars represent CNVs. The green line represents the mother, the red line the father and the blue line the proband. Three CNVs were found in the proband on chromosomes 2, 14 and 16, Two were paternally inherited (chromosomes 2 and 16) and the third was present in all three individuals tested. Additional file 2: Table S5. Fibroblast gene expression levels.
