# Supplementary material for: Language impairment in a case of a complex chromosomal rearrangement with a breakpoint downstream of FOXP2
Source: Mol Cytogenet. 2015 Jun 10;8:36. doi: 10.1186/s13039-015-0148-1 (PMC4460787; doi:10.1186/s13039-015-0148-1)
Supplement: Additional file 1: — Table S1. Proband scores at 9;6 (9 years and 6 months of biological age) for The Illinois Test of Psycholinguistic Aptitudes. Table S2. All BACs hybridized to patient sample to map breakpoint location. The BACs localization in the patient is highlighted in color (e.g., the derivative 11p in blue, and the derivative 7p in yellow). Table S3. Average CT values across three RNA batches. In parentheses, SE, standard error. Table S4. Relative expression levels of FOXP2 in all family members compared to the proband, measured with Taqman probes. Figure S1. A Typical Scores of the proband aged 10;8. Subtests where answers were required using language are classified as Verbal. Subtests where language was not required to provide answers are marked Manipulative. Typical Scores within 8-12 are normal. B. Typical Verbal Scores of the proband. Typical Verbal Scores refer to subtests where answers are required in Castilian-Spanish or Valencian. Typical Scores within 8-12 values are normal. Figure S2. FISH on metaphase spreads from the proband. Each panel shows the hybridization signal (green) from a single BAC probe, as indicated (the library name has been omitted). The chromosomes are counterstained in DAPI, blue. In each panel a white arrow identifies the normal chromosome 7 (where present), a green arrow the derivative 7, a yellow arrow the normal chromosome 11 (where present), and a red arrow the derivative 11. Figure S3. CNVs were mapped in the probands and parents using Affymetrix Cytoscan and copy number changes were called within Affymetrix Chromosome Analysis Suite (ChAS). Stars represent CNVs. The green line represents the mother, the red line the father and the blue line the proband. Three CNVs were found in the proband on chromosomes 2, 14 and 16, Two were paternally inherited (chromosomes 2 and 16) and the third was present in all three individuals tested. [file 13039_2015_148_MOESM1_ESM.docx]

**Additional file 1**

**SUPPLEMENTARY TABLES**

**Supplementary Table 1** Proband scores at 9;6 (9 years and 6 months of biological age) for The Illinois Test of Psycholinguistic Aptitudes.

DS=Direct Score, PA= Psycholinguistic Age and TS= Typical Score.

|  | **DS** | **PA** | **TS** |
| --- | --- | --- | --- |
| Auditory comprehension | 27 | 6;0 | 28 |
| Visual Comprehension | 24 | 7;5 | 31 |
| Sequential Visuo-motor memory | 13 | 4;8 | 30 |
| Auditory Association | 26 | 7;9 | 30 |
| Sequential Auditory memory | 10 | 7;3 | 32 |
| Visual association | 28 | 7;10 | 32 |
| Visual Integration | 38 | 6;10 | 28 |
| Verbal Expression | 40 | 7;2 | 28 |
| Grammatical Integration | 15 | 5;8 | 24 |
| Motor expression | 20 | 6;10 | 29 |
| **TOTAL** | 241 | 6;7* | 29** |

*the proband Psycholinguistic Age is of 6 years and 7 months at 9 years and 6 months of biological age;

**the average score at this biological age is 36, with 52 being the maximum.

**Supplementary Table 2** All BACs hybridized to patient sample to map breakpoint location. The BACs localization in the patient is highlighted in color (e.g., the derivative 11p in blue, and the derivative 7p in yellow).

| **BAC** | **Start** | **End** | **Cytogenetic band** | **Patient** |
| --- | --- | --- | --- | --- |
| CTB-164D18 | 255,000 |  | 7p tel | derivative 11p tel |
| CTB-146A2 | 4,537,635 | 4,634,001 | 7p22.1 | derivative 11p |
| RP11-352E12 | 10,082,487 | 10,276,028 | 7p21.3 | derivative 11p |
| RP11-323K15 | 17,790,338 | 17,964,707 | 7p21.1 | derivative 11p |
| RP11-71F18 | 19,436,044 | 19,620,962 | 7p21.1 | derivative 11p |
| RP11-486P11 | 20,076,654 | 20,182,871 | 7p21.1 | derivative 11p |
| RP11-257D9 | 20,355,296 | 20,523,106 | 7p21.1 | derivative 11p |
| RP11-61N24 | 20,748,162 | 20,919,479 | 7p21.1-p15.3 | derivative 11p |
| RP11-799L23 | 20,810,041 | 21,001,537 | 7p21.1-p15.3 | split |
| RP11-97L4 | 20,954,043 | 21,104,475 | 7p15.3 | split |
| RP11-1129E15 | 21,042,192 | 21,208,991 | 7p15.3 | derivative 7q |
| RP11-211J15 | 21,207,376 | 21,291,073 | 7p15.3 | derivative 7q |
| RP11-445O1 | 21,621,709 | 21,702,517 | 7p15.3 | derivative 7q |
| RP11-343P21 | 24,523,090 | 24,692,665 | 7p15.3 | derivative 7q |
| RP5-1091E12 | 55,247,443 | 55256627 | 7p11.2 | derivative 7q |
| **CEN** |  |  |  |  |
| RP11-88E13 | 70134944 | 70306652 | 7q11.22 | derivative 7p |
| RP11-84L12 | 82817474 | 83001602 | 7q21.11 | derivative 7p |
| RP11-118M19 | 91564571 | 91727157 | 7q21.2 | derivative 7p |
| CTB-104I4 | 92,640,907 | 92,832,097 | 7q21.2-21.3 | derivative 7p |
| RP11-49N15 | 92,862,849 | 93,012,161 | 7q21.3 | derivative 7p |
| CTD-2007G21 | 93,486,290 | 93,591,077 | 7q21.3 | derivative 7p |
| RP11-1062K14 | 93,555,485 | 93,770,700 | 7q21.3 | derivative 7p |
| RP11-7B9 | 93,714,463 | 93,884,065 | 7q21.3 | derivative 7p |
| RP11-248I13 | 93,933,453 | 94,094,489 | 7q21.3 | derivative 11p |
| RP11-457A22 | 94039268 | 94193482 | 7q21.3 | derivative 11p |
| RP11-186B5 | 103039620 | 103219136 | 7q22.1 | derivative 11p |
| RP11-74B7 | 103361025 | 103531233 | 7q22.1 | derivative 11p |
| RP11-340M13 | 107685827 | 107845165 | 7q31.1 | derivative 11p |
| RP11-54P11 | 107687613 | 1077879900 | 7q31.1 | derivative 11p |
| RP11-117I4 | 113695289 | 113876135 | 7q31.1 | derivative 11p |
| RP11-65M11 | 113823906 | 113996140 | 7q31.1 | derivative 11p |
| RP11-378F9 | 113995975 | 114169064 | 7q31.1 | derivative 11p |
| RP11 65C13 | 114053980 | 114186040 | 7q31.1 | derivative 11p |
| RP11 25C3 | 114217577 | 114374043 | 7q31.1 | derivative 11p |
| RP11-259A16 | 114374055 | 114528369 | 7q31.1-31.2 | derivative 11p |
| RP11-1N24 | 114451504 | 114603837 | 7q31.1-31.2 | Split, derivative 7q, derivative 11p |
| RP11-243D16 | 114556605 | 114733501 | 7q31.1-31.2 | derivative 7q |
| RP11-103A1 | 114815067 | 114979199 | 7q31.2 | derivative 7q |
| RP11-17M12 | 115135991 | 115295452 | 7q31.2 | derivative 7q |
| RP11-383J9 | 115454578 | 115633765 | 7q31.2 | derivative 7q |
| RP11-242J21 | 115615548 | 115799625 | 7q31.2 | derivative 7q |
| RP11-51M22 | 115841068 | 116003386 | 7q31.2 | derivative 7q |
| RP11-60D5 | 155229982 | 155394379 | 7q36.3 | derivative 7q |
| CTB-3K23 | 157425793 |  | 7q tel | derivative 7q tel |
| **Chromosome 11** | | | | |
| **BAC** | **Start** | **End** | **Cytogenetic band** | **Patient** |
| CTC-908H22 | 125000 |  | 11p tel | der 7p tel |
| WI2-1783D03:G248P85929B2 | 5209658 | 5249744 | 11p15.4 | der 7p |
| WI2-2195C14:G248P87010B7 | 5228003 | 5265607 | 11p15.4 | der 7p |
| RP11-366N23 | 31647249 | 31805088 | 11p13 | der 7p |
| RP4-607I7 | 35029913 | 35183987 | 11p13 | der 7p |
| RP1-68D18 | 35189741 | 35373422 | 11p13 | der7p |
| RP1-136N16 | 35,183,888 | 35,189,840 | 11p13 | der7p |
| RP11-115P8 | 35700817 | 35854414 | 11p13 | der7p |
| RP11-194C13 | 36,714,722 | 36,894,955 | 11p12 | der7p |
| RP11-324K6 | 37,717,135 | 37,790,446 | 11p12 | der7p |
| RP11-1101H5 | 37,982,581 | 38,169,205 | 11p12 | der7p |
| RP11-78B7 | 38,361,664 | 38,533,287 | 11p12 | der7p |
| RP11-63D14 | 38,455,222 | 38,604,308 | 11p12 | der7p |
| RP11-81O6 | 38,601,145 | 38,749,858 | 11p12 | split |
| RP11-99H2 | 38,621,572 | 38,794,051 | 11p12 | der 11p |
| RP11-89G12 | 38,716,362 | 38,880,635 | 11p12 | der 11p |
| RP11-1036E20 | 58672511 | 58895398 | 11q12.1 | der 11q |
| RP11-796A5 | 88909028 | 89056388 | 11q14.3 | der 11q |
| RP11-141H6 | 88910581 | 89055610 | 11q14.3 | der 11q |

**Supplementary Table 3**  Average C_T_ values across three RNA batches. In parentheses, SE, standard error.

| **Average C_T_** | **Proband**  **(SE)** | **Sibling**  **(SE)** | **Mother**  **(SE)** | **Father**  **(SE)** |
| --- | --- | --- | --- | --- |
| *FOXP2* (Hs01074134_m1) | 29.83 (0.18) | 30.39 (0.40) | 29.91 (0.32) | 29.96 (0.29) |
| *FOXP2* (Hs01081804_m1) | 28.97 (0.50) | 29.32 (0.30) | 28.09 (0.36) | 29.59 (0.16) |
| *IPO8* (Hs00183533_m1) for Hs01074134_m1 | 27.95 (0.12) | 28.12 (0.35) | 28.24 (0.31) | 28.42 (0.15) |
| *IPO8* (Hs00183533_m1) for Hs01081804_m1 | 30.66 (0.72) | 29.29 (0.31) | 29.20 (0.21) | 29.90 (0.21) |

As this was a duplex qPCR, the assay for housekeeping gene (*IPO8*) was used twice, with each *FOXP2* assay. In the statistical comparison of the proband to each family member separately with the Mann-Whitney-Wilcoxon test, all p-values are > 0.05.

**Supplementary Table 4** Relative expression levels of *FOXP2* in all family members compared to the proband, measured with Taqman probes.

| **Expression compared to proband** | **Proband** | **Sibling** | **Mother** | **Father** |
| --- | --- | --- | --- | --- |
| *FOXP2* (Hs01074134_m1) | 1 | 1.010461 | 1.034541 | 0.9390217 |
| *FOXP2* (Hs01081804_m1) | 1 | 0.859642 | 0.772752 | 0.8859205 |

**SUPPLEMENTARY FIGURE LEGENDS**

**Supplementary Figure 1** A Typical Scores of the proband aged 10;8. Subtests where answers were required using language are classified as Verbal. Subtests where language was not required to provide answers are marked Manipulative. Typical Scores within 8-12 are normal. B. Typical Verbal Scores of the proband. Typical Verbal Scores refer to subtests where answers are required in Castilian-Spanish or Valencian. Typical Scores within 8-12 values are normal

**Supplementary Figure 2** FISH on metaphase spreads from the proband. Each panel shows the hybridization signal (green) from a single BAC probe, as indicated (the library name has been omitted). The chromosomes are counterstained in DAPI, blue. In each panel a white arrow identifies the normal chromosome 7 (where present), a green arrow the derivative 7, a yellow arrow the normal chromosome 11 (where present), and a red arrow the derivative 11.

**Supplementry Figure 3** CNVs were mapped in the probands and parents using Affymetrix Cytoscan and copy number changes were called within Affymetrix Chromosome Analysis Suite (ChAS). Stars represent CNVs. The green line represents the mother, the red line the father and the blue line the proband. Three CNVs were found in the proband on chromosomes 2, 14 and 16, Two were paternally inherited (chromosomes 2 and 16) and the third was present in all three individuals tested.

**Supplementary Figure 1**


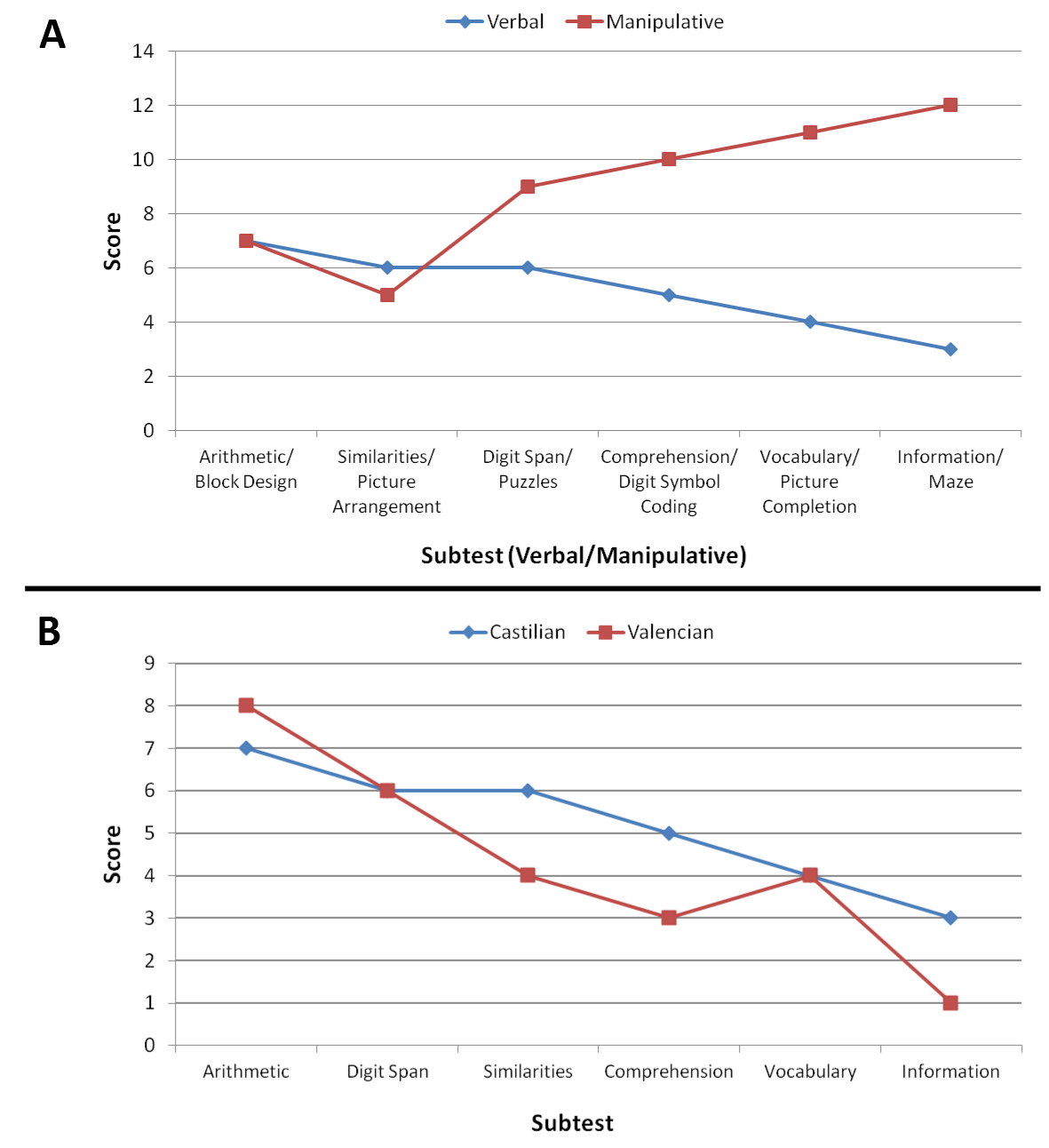


**Supplementary Figure 2**

**
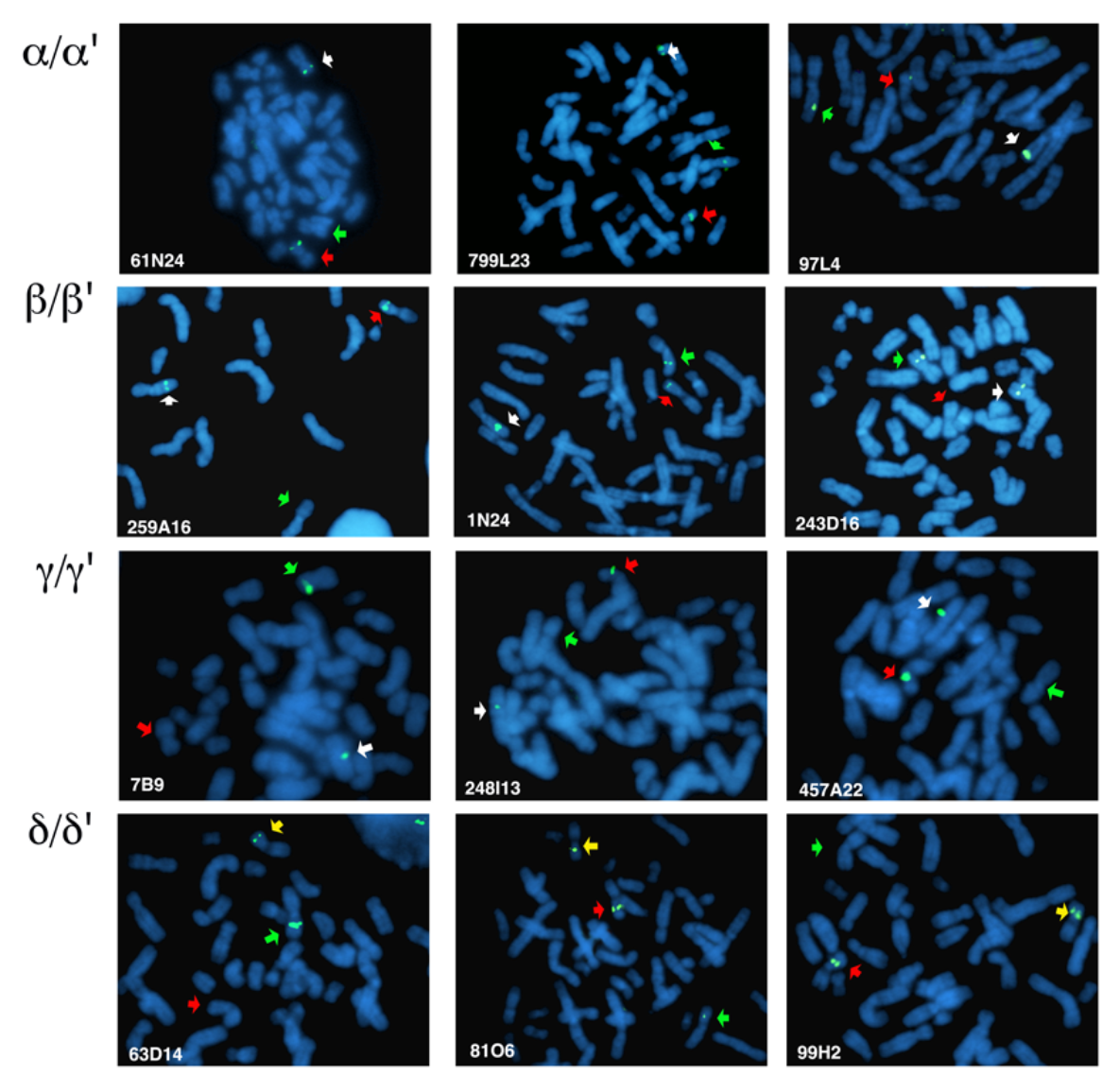
**

**Supplementary Figure 3**


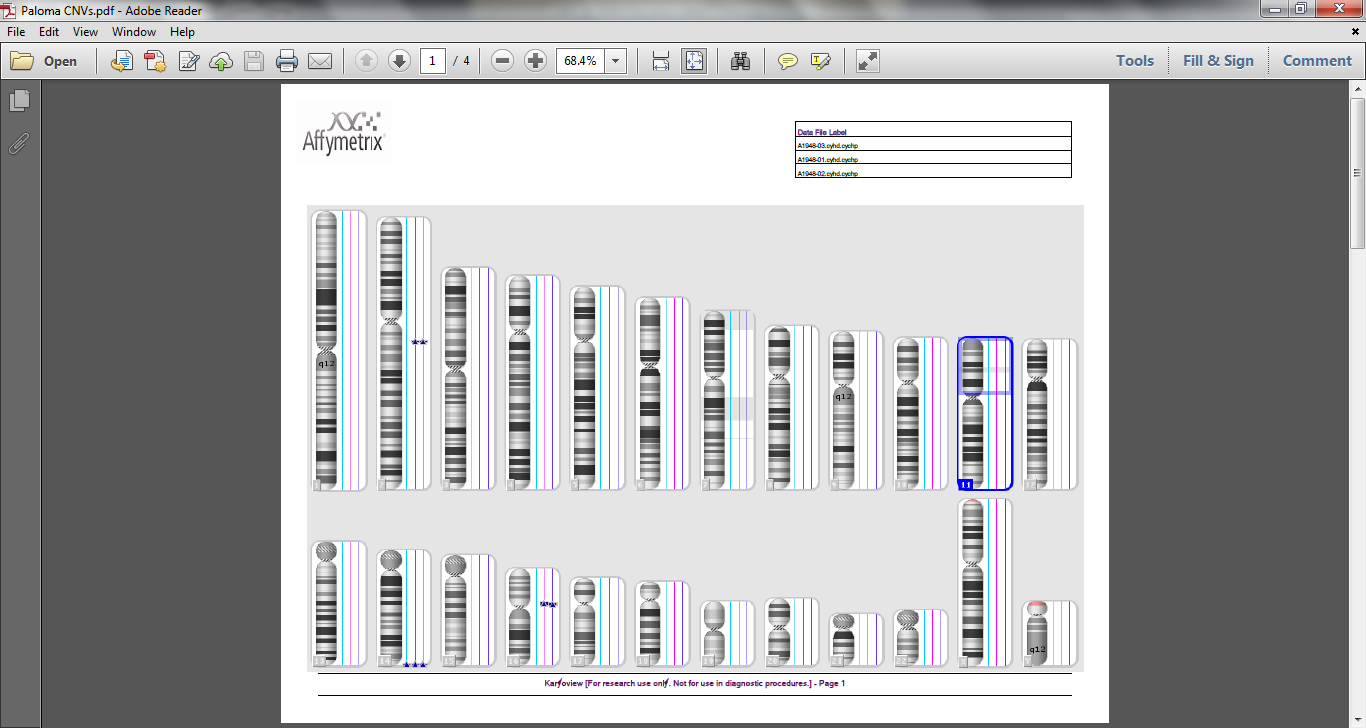


­­­
